# Choosing the proper path: outcomes of subxiphoid vs. lateral intercostal approaches in the resection of anterior mediastinal masses

**DOI:** 10.3389/fsurg.2024.1463881

**Published:** 2024-12-03

**Authors:** Xuechun Leng, Mengzou Chen, Yang Zhang, Jian Gao, Zhenbing You, Zhongwu Hu

**Affiliations:** ^1^ Department of Thoracic Surgery, The Affiliated Huaian No.1 People’s Hospital, Nanjing Medical University, Huai'an, Jiangsu, China; ^2^ Department of Anesthesiology, The Affiliated Huaian No.1 People’s Hospital, Nanjing Medical University, Huai'an, Jiangsu, China; ^3^ Clinical Statistics Center, The Affiliated Huaian No.1 People’s Hospital, Nanjing Medical University, Huai'an, Jiangsu, China

**Keywords:** lateral intercostal approach, video-assisted thoracoscopic surgery (VATS), anterior mediastinal mass, short-term efficacy, subxiphoid approach

## Abstract

**Background:**

While the subxiphoid approach (SA) in thoracoscopic thymectomy offers benefits in terms of fat removal and pain reduction, it remains unclear which patients with anterior mediastinal masses benefit most from the subxiphoid vs. the lateral intercostal approach (LA).

**Methods:**

This retrospective study analyzed patients treated for anterior mediastinal masses at our center from January 2019 to December 2023. Patients were categorized into two groups based on the surgical approach: SA (35 cases) and LA (56 cases). Demographic data, clinical characteristics, perioperative metrics, and short-term outcomes were compared.

**Results:**

Ninety-one patients were included, with diagnoses including thymic cysts (43), thymomas types A, AB, and B1 (24), B2 thymomas (18), thymic carcinoma (6).No significant differences were found between the groups in terms of gender, age, tumor size, body mass index, conversion to sternotomy, or blood loss. The LA group, however, experienced shorter surgical durations (*P* < 0.001), less drainage (*P* = 0.021), shorter hospital stays (*P* < 0.001), and lower hospitalization costs (*P* = 0.024). Pain scores on the visual analogue scale were similar between groups on the day of surgery and the first postoperative day.

**Conclusion:**

The findings suggest that the lateral intercostal approach is preferable for patients with thymic cysts and Masaoka stage I–II thymomas without myasthenia gravis due to its efficiency and cost-effectiveness.

## Introduction

The detection of anterior mediastinal masses has significantly increased due to the widespread use of computed tomography screening (1). Surgical intervention remains the primary treatment strategy for these masses. Video-assisted thoracoscopic surgery (VATS) offers several advantages over traditional sternotomy, including reduced trauma, minimal bleeding, decreased pain, quicker recovery, shorter hospital stays, and minimal impact on cardiopulmonary function, all while maintaining comparable recurrence and survival rates (2–4). Among the VATS techniques, the classic lateral intercostal approach (LA) and the subxiphoid approach (SA) are prevalent. Increasing evidence suggests that the SA provides superior exposure, facilitates bilateral phrenic nerve clearance and fat removal, and offers a visual field akin to that of sternotomy, potentially reducing postoperative pain and skin numbness (5, 6). Despite its growing preference among thoracic surgeons, clear indications for choosing between the SA and LA for anterior mediastinal mass resections remain undefined. This retrospective study compares clinical data from patients undergoing LA and SA at our center, aiming to delineate these indications.

## Methods

### Study cohort and clinical data

We retrospectively reviewed all patients who underwent VATS resection for anterior mediastinal tumors at our center from January 2019 to December 2023. Inclusion criteria included: (I) adults (≥18 years of age); (II) diagnosis of an anterior mediastinal tumor; and (III) complete clinical data and computed tomography scans available. Exclusion criteria were: (I) patients who underwent median sternotomy; (II) lateral position intercostal chest insertion; (III) malignant tumors with apparent organ invasion; (IV) pericardial cysts; and (V) extensive thoracic adhesions. We documented patient characteristics and staged thymomas according to the Masaoka classification (7).

### Surgical technique

#### Subxiphoid approach (SA)

**Figure 1:**
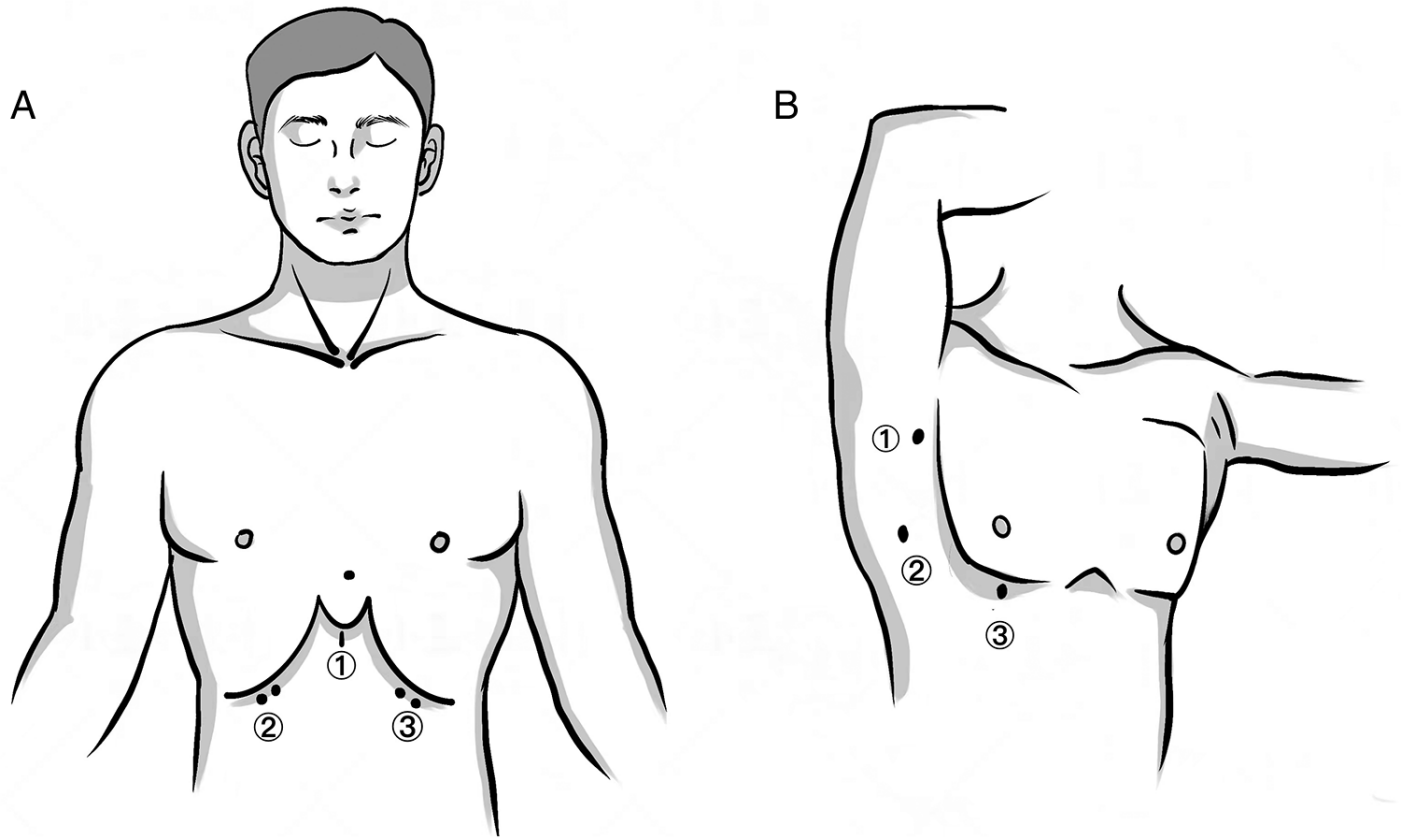
Skin incisions of the two approaches. **(A)** SA: (1) a 3 cm transverse or vertical incision under the xiphoid process as the observational port; (2, 3) a 0.5–1 cm incision at the intersection of the bilateral midclavicular line and the costal arch as the operating port. **(B)** LA: (1) a 0.5 cm incision at the anterior axillary line in the third intercostal space as the operating port; (2) a 1 cm incision at the midaxillary line in the fifth intercostal space as the observational port; (3) a 1 cm incision at the mid-clavicular line in the fifth intercostal space as the operating port.

Patients were placed in a lithotomy position. A 3 cm transverse or vertical incision was made under the xiphoid process for thoracoscope insertion. Additional 0.5 cm incisions were made at the bilateral midclavicular line and costal arch junction for trocar placement. We created an artificial pneumothorax (CO2 pressure: 8–10 mmHg) and performed mediastinal dissection down to the left innominate vein using an ultrasonic knife. Thymic veins were severed and the specimen was removed through the xiphoid incision. Thoracic drainage utilized 15F or 22F negative pressure balls (Figure 1A).

#### Lateral intercostal approach (LA) (Supplementary Video S1)

Patients were positioned supinely. Incisions were made in the third intercostal space at the anterior axillary line, and in the fifth intercostal space at the mid-clavicular and mid-axillary lines. Following pneumothorax induction (CO2 pressure: 8–10 mmHg), resection was performed predominantly from the right side, unless the tumor was left-dominant. The bilateral LA approach facilitated complete adipose tissue removal in patients with myasthenia gravis or positive anti-acetylcholine receptor antibodies (Figure 1B). Based on the size of masses, extended the ③ incision to 1.5–2 cm (Figure 1B), and used a retrieval bag to remove the specimen.

### Analgesic strategies

We employed multimodal analgesia including paravertebral, intercostal nerve block, or incision infiltration. A patient-controlled analgesia (PCIA) pump delivered a mix of 100 μg sufentanil and 100 μg dexmedetomidine in 100 ml saline at 2 ml/h. Additional NSAIDs or opioids were administered as needed, and pain levels were assessed using the visual analogue scale (VAS) on the day of and the day after surgery.

### Ethical statement

The study adhered to the ethical standards of the Declaration of Helsinki (2013 revision) and was approved by the Ethics Committee of Huai'an First People's Hospital, Nanjing Medical University. Consent for this retrospective analysis was waived.

### Statistical analysis

Statistical analyses were performed using SPSS (version 21.0; IBM SPSS Statistics, Chicago, IL, USA). Continuous variables are expressed as mean ± SD, and categorical variables as frequencies and percentages. Differences were evaluated using independent *t*-tests or Wilcoxon rank-sum tests for continuous variables and chi-square tests for categorical variables. A *p*-value < 0.05 was considered statistically significant.

## Results

### Patient demographics and baseline characteristics

Our study included 91 patients: 35 underwent the subxiphoid approach (SA) and 56 underwent the lateral intercostal approach (LA). No significant differences were found between the two groups in age, gender, body mass index, and tumor size (all *P* > 0.05). Thymic cysts were diagnosed in 47.3% of all patients, with a higher incidence in the SA group compared to the LA group, which had a higher proportion of types A, AB, and B1 thymomas (*P* < 0.05). Myasthenia gravis (MG) was present in 6.6% of the patients (Table 1).

**Table 1:** Demographic and clinical characteristics of the two groups.

Variables	SA	LA	*P*
Demographics			
Age (mean, years)	55.11 ± 8.81	55.27 ± 10.33	0.092
Male/Female	14/21	22/34	0.096
BMI (kg/m^2^)	24.84 ± 3.6	25.36 ± 3.9	0.452
MG, *n* (%)	3 (8.6)	3 (5.4)	0.548
Tumor size (diameter, cm)	3.76 ± 1.27	3.8 ± 1.43	0.85
Comorbidities, *n* (%)			
Diabetes	4	3	0.290
Hypertension	10	13	0.567
Coronary heart disease	2	4	0.789
Tumor type, *n* (%)			0.63
Thymic cyst	22 (62.9)	21 (37.5)	<0.05
Thymoma (A, AB, B1)	5 (14.3)	19 (33.9)	*P* < 0.05
Thymoma (B2)	5 (14.3)	13 (33.9)	
Thymic cancer	3 (8.6)	3 (5.4)	
Thymoma masaoka stage, *n* (%)	13 (37.1)	35 (62.5)	0.882
I	4	10	
II	9	25	

Continuous variables are presented as the mean ± SD. Categorical variables are presented as *n* (proportions). SA, subxiphoid approach thoracoscopic thymectomy; LA, lateral intercostal approach thoracoscopic thymectomy; BMI, body mass index; MG, myasthenia gravis; SD, standard deviation.

### Short-term outcomes

Operative outcomes varied significantly between the two approaches. The LA group benefited from shorter operative times (*P* = 0.00), reduced hospital stays (*P* = 0.00), and less chest tube drainage (*P* = 0.007) compared to the SA group. Pain levels assessed by the Visual Analogue Scale (VAS) showed no difference on the day of surgery or the first postoperative day. Additionally, the use of extra analgesics post-surgery did not differ significantly between the groups.

Postoperative complications were minimal and comparable between the groups, including phrenic nerve paralysis, effusions, pneumothoraces requiring thoracocentesis, secondary operations, and dyspnea (all *P* > 0.05). Notably, one patient in the LA group underwent a second surgery due to persistent hemothorax, and one patient in the SA group developed a pleural effusion. Financial considerations also differed, with the SA group incurring higher hospitalization costs than the LA group (*P* = 0.024) (Table 2).

**Table 2:** Perioperative data and in-hospital outcomes.

Variables	SA	LA	*P*
Operation time (min)	95.57 ± 32.44	61.79 ± 28.1	0.00
Conversion to sternotomy, *n* (%)	1 (2.9)	2 (3.6)	0.853
Blood loss (ml)	10–400	10–1000	0.923
Postoperative hospital stays (d)	6.31 ± 1.98	4.21 ± 1.39	0.00
Chest tube drainage (ml)	75–1,800	0–760	0.007
VAS			
On the day of surgery	0–6	0–6	0.96
On the first day after surgery	2–6	2–6	0.75
Extra analgesics, *n* (%)	3 (8.6)	8 (14.3)	0.416
In-hospital death, *n* (%)	0	0	N/A
Complications, *n* (%)			
Phrenic nerve paralysis	0	0	N/A
Effusion/pneumothorax requiring			
Thoracocentesis	1	0	0.203
Secondary operation	0	1	0.427
Dyspnea	0	0	N/A
Hospitalization cost, CNY (China Yuan)	33,193 ± 5,680	29,852 ± 7,339	0.024

Continuous variables are presented as the mean ± SD. Categorical variables are presented as *n* (proportions). Continuous variables were assessed by an independent sample *t*-test or Wilcoxon rank-sum test and categorical variables were compared using a chi-square test. VAS, visual analogue scale; SD, standard deviation.

## Discussion

Surgical resection stands as the primary treatment for anterior mediastinal masses. Since the introduction of VATS thymectomy in 1993 by a team in Boston, MA, USA, the intercostal approach has been globally recognized and adopted (8, 9). Despite its widespread use, the lateral intercostal approach (LA) encounters limitations, particularly in accessing the contralateral phrenic nerve and reaching the superior pole of the thymus. Moreover, intercostal incisions are known to potentially damage nerves, leading to chronic neuralgia and abnormal skin sensations (10). Nevertheless, the five-year survival rates for patients with Masaoka stage I-II thymomas undergoing this procedure align closely with those seen in open surgery (11).

In 1999, Kido introduced the subxiphoid approach (SA) as an alternative for resecting anterior mediastinal masses (12). Presently, both single- and three-port subxiphoid approaches are employed (13), though they come with their challenges, particularly in obese patients or those with mediastinal stenosis where increased chest rigidity complicates the procedure. The SA often necessitates additional surgical instruments and is especially beneficial in larger masses when combined with sternal elevation techniques (14, 15). Mao et al. utilized a sternal elevation device along with a closed incision protection sleeve and CO2 pressurization to enhance the operable space behind the sternum, facilitating the resection of larger or more complex mediastinal tumors (14).

Contrary to earlier findings, our study indicates no significant benefits of SA over LA regarding surgical duration, hospital stay, or drainage volume. While some research shows no difference in operative times between the two approaches (16), others have reported shorter times for SA (17, 18). Contrasting with previous reports that averaged 120 min for LA (13), our findings demonstrate a notably shorter average operating time of 61.79 ± 28.1 min for LA, which might be attributed to the efficient surgical techniques employed, such as supine positioning, single-lumen endotracheal intubation, and a closed incision with a protective sleeve to prevent CO2 leakage. This streamlined process could potentially contribute to incomplete fat clearance and reliance on a unilateral approach differed from the bilateral LA approach in the Madhuri's study (19). Additionally, a shorter operating time inherently reduces the risk of complications (20) and could be influenced by the SA's steep learning curve, as Suda noted it requires 30–40 cases to achieve proficiency (21).

Pain management, evaluated through Visual Analog Scale (VAS) scores, showed no significant differences postoperatively, aligning with findings from Wang et al., where early postoperative pain improvements in SA did not extend beyond the early recovery phase (22). The use of multimodal analgesia and advanced drainage techniques likely contributed to the consistently low pain levels observed.

In our cohort, three patients with Myasthenia Gravis (MG) were treated under each approach, highlighting the importance of removing ectopic thymic tissue to alleviate symptoms and reduce crises (23, 24). Thymectomy and removal of fat alleviates the symptoms of MG and reduces the occurrence of myasthenic crises, which has become an international consensus (10, 25). Studies have shown that minimally invasive procedures can effectively manage MG symptoms as well as traditional sternotomy (26, 27), with SA potentially offering superior outcomes due to its ability to ensure a more complete thymectomy and fat clearance (18, 28).

The incidence of requiring a conversion to sternotomy was similar between the groups, primarily due to injuries to the innominate vein. Both approaches demonstrated a low 30-day postoperative complication rate with no significant differences observed. Cost analysis revealed that LA was more economically advantageous compared to SA, reflecting the latter's requirement for more specialized equipment and potentially longer operating times.

In our single-center study, the high prevalence of thymic cysts (47.3%) underscores the necessity for precise surgical intervention. MRI, which was not utilized in all cases at our center, could provide detailed lesion characterization, potentially preventing unnecessary surgeries (29, 30). In contrast, acquired thymic cysts are much more common, tend to be multilocular, and may arise in association with neoplasms such as thymomas, lymphomas, or germ cell tumors (30).

Our findings suggest that while SA offers a comprehensive solution akin to sternotomy for non-MG thymoma patients, it does not significantly impact long-term survival compared to LA (26, 31–33). The choice between SA and LA should, therefore, be tailored based on individual patient profiles and available resources.

Despite the strengths of this retrospective study, its limitations include potential selection bias, a small sample size, and the single-center design, which might affect the generalizability of the results. Additionally, the lack of long-term follow-up restricts our ability to assess the durability of these surgical outcomes. Future multicenter studies with larger cohorts and longer follow-ups are essential to further elucidate the optimal surgical approach for resecting anterior mediastinal masses.

## Conclusions

While the SA is favored by many surgeons for its theoretical benefits in visualization and reduced pain, our findings and those of other recent studies suggest that the choice between SA and LA should be guided more by specific patient anatomical considerations, surgeon expertise, and resource availability rather than inherent superiority of one technique over the other. For patients with thymic cysts and early-stage thymomas without MG, LA may offer a more efficient and cost-effective option.

## Data Availability

The original contributions presented in the study are included in the article/Supplementary Material, further inquiries can be directed to the corresponding author.
